# Biophysical reviews top five: atomic force microscopy in biophysics

**DOI:** 10.1007/s12551-021-00820-x

**Published:** 2021-07-10

**Authors:** Toshio Ando

**Affiliations:** Nano Life Science Institute (WPI-NanoLSI), Kanazawa University, Kakuma-machi, Kanazawa, 920-1192 Japan

## Abstract

Since its invention in the late 1980s, atomic force microscopy (AFM), in which a nanometer-sized tip is used to physically interrogate the properties of a surface at high resolution, has brought about scientific revolutions in both surface science and biological physics. In response to a request from the journal, I have prepared a top-five list of scientific papers that I feel represent truly landmark developments in the use of AFM in the biophysics field. This selection is necessarily limited by number (just five) and subjective (my opinion) and I offer my apologies to those not appearing in this list.

The technique of atomic force microscopy (AFM) constitutes a multifunctional toolbox in modern biophysics (see reviews: Müller and Dufrêne 2008; Ando et al. 2014; Dufrêne et al. 2017; Valotteau et al. 2019). Using AFM, we can perform various measurements under physiological conditions that are infeasible with other techniques: e.g., dynamic high-resolution imaging of biomolecules during their functional activity, recognition and localization of specific molecules, force measurements to estimate the strength of intra- and intermolecular bonds at the single-molecule level, and the site-specific recording of the elasticity of biological surfaces. This current metrological prosperity can, of course, be traced back to its point of origin (Binnig et al. 1986) but it also owes a great deal to many technical developments and creative studies carried out since then.

In terms of technical advances, although all AFM experiments utilize similar instrumental components, for biological/biophysical applications AFM devices must be operated in a particular manner best suited to the peculiarities of the sample (e.g., typically soft, hydrated/in solution, and highly dynamic). In this regard, AFM instruments that are geared to biophysical measurements are typically operated in the AC mode where the cantilever is oscillated at its resonant frequency in solution. Each of these technical components, necessary to achieve this specialization, has its own origins: e.g., Binnig et al. 1987 for cantilevers, Meyer and Amer 1988 for the optical beam deflection technique able to measure cantilever deflection, Zhong et al. 1993 for the AC mode, and Marti et al. 1987 for in-liquid imaging. Such technical and instrumental advances made possible a number of groundbreaking biophysical studies (Drake et al. 1989; Weisenhorn et al. 1993; Butt et al. 1993; Florin et al. 1994; Hinterdorfer et al. 1996) in which core principles and techniques were established and which have gone on to form the basis of much of modern AFM-based biophysical research. It is from these pioneering studies that I have made my top five selection (Table 1). Although the actual results produced from these pioneering studies may not be necessarily particularly refined, they have all opened new avenues for studying different aspects of biological samples with AFM. In the next section, I offer a short description of these studies along with a brief justification of their selection in this list.

**Table 1:** Five selected studies

Drake et al. 1989	The first attempt to visualize dynamic biomolecular processes
Weisenhorn et al. 1993	The first quantification of elasticity of living cells
Butt et al. 1993	The first consideration of scan speed limit in AFM
Florin et al. 1994	The first attempt to measure adhesion forces between individual ligand-receptor pairs
Hinterdorfer et al. 1996	The first attempt of recognition imaging

## Top five list justification

Very soon after the advent of AFM, Paul Hansma and his colleagues observed, at ~1-min intervals, the clotting process by fibrin molecules following the digestion of fibrinogen with thrombin (Drake et al. 1989). This study inspired exploration into the potential of AFM for the observation of dynamic biological processes with a few such examples being antibody-antigen binding (Ohnesorge et al. 1992), live cells infected by viruses (Häberle et al. 1992), DNA bending upon binding to λ Cro protein (Erie et al. 1994), DNA digestion with DNase (Bezanilla et al. 1994), DNA–RNA polymerase binding process (Guthold et al. 1994), RNA replication reaction (Kasas et al. 1997), and the diffusion of RNA polymerase along a DNA strand (Guthold et al. 1999). One of the inventors of AFM, Gerd Binnig, said, “In biology, use of the force microscope will probably become quite common because of its ability to deliver films of processes” (Binnig 1992). However, at the time it took at least 30 s to capture an AFM image. In this regard, I point out that high-speed AFM capable of filming at ~10 frames per second was established 16 years later (Ando et al. 2008).

AFM-based mechanobiological measurements are now widely performed, in particular for living normal and cancerous cells (see reviews: Krieg et al. 2019; Runel et al. 2021). The elasticity (Young’s modulus) of living cells was first quantified to be 0.013–0.15 MPa from the force-versus-indentation curve measurements on a lung carcinoma cell (Weisenhorn et al. 1993). In that study, the measured value of Young’s modulus for the cancer cell was significantly smaller than that recorded for normal cells, a finding which is now well accepted. Moreover, metastatic tumor cells are more than 70% “softer” compared with benign tumor cells (Xu et al. 2012). Thus, this initial study by Weisenhorn et al. (1993) established the basis of using AFM as a diagnostic tool.

The study by Butt et al. (1993) was the first to provide a theoretical consideration for a scan speed limit in AFM imposed by the cantilever’s dynamic response. This study did not intend to propose or develop faster AFM but just quantified the scan speed limit in the constant height DC mode AFM based on available (at that time) cantilever properties. Nonetheless, beyond the authors’ intention, this study likely influenced the development of high-speed AFM that was initiated in 1993. A few years later, prototypic HS-AFM systems and acquired molecular movies were reported (Vianni et al. 2000; Ando et al. 2001).

Gaub and colleagues were the first to quantify the adhesive force of a single protein-ligand bond using AFM for the biotin-avidin pair (Florin et al. 1994). A “bait molecule” (avidin) was attached to the biotin-coated cantilever tip, while the sample (an agarose bead) was biotinylated. Then, unbinding forces were measured from force-versus-distance (FD) curves, in a pulling process somewhat like fishing. Later, this study was refined by changing the rate of force application, allowing the quantification of the energy landscape of the biotin-avidin interaction (Yuan et al. 2000), based on the Bell-Evans model (Bell 1978; Evans and Ritchie 1997). This force spectroscopy method has been widely applied to various biomolecular interactions and unfolding of proteins (see Review: Puchner and Gaub 2009). Moreover, the rate of force application has now been enhanced to the level comparable to molecular dynamics simulations (Rico et al. 2013).

Biological surfaces, such as membrane surfaces, often contain a diverse range of proteins and other biomolecules. In such a case, it is difficult to specify and localize individual species from a topography image alone. In the first attempt to detect and localize a specific type of species, the just described “fishing-like” experiment (Florin et al. 1994) was extended to two-dimensional measurements (Hinterdorfer et al. 1996), where bait molecules were covalently attached to the cantilever tip via a flexible linker. To overcome the time-consuming FD curve measurements, a fast method called “TREC imaging” was later invented (Stroh et al. 2004) in which the cantilever’s upward and downward swings are separately detected for simultaneous capturing of recognition and topography images, respectively. This type of recognition imaging has been used to localize receptors and ion channels on cell surfaces (see Review: Chtcheglova and Hinterdorfer 2018) and specific binding sites in protein and DNA molecules (Zhu et al. 2010).

## Concluding remarks

It was very difficult to limit my selection to only five papers among the numerous great works within the AFM field but this was the brief given with the invitation to write this top-five list. Rather than scientific quality, I have set a high value on pioneering studies that have influenced or inspired subsequent studies. I apologize to all whose works have not been selected in the top-five format. There are many great pioneering studies showing beautiful high-resolution AFM images of membrane proteins but it was impossible to select one because of multiple papers published in the same year (1990). Moreover, I could have included, but did not, many excellent AFM studies on mapping of material properties such as flexibility, electrostatic potential, and adhesiveness.

It is hoped that this list may be useful to those just starting out in the bio-AFM field wishing to have some advice on important historical papers from the perspective of a colleague who has been working in this area for a long time.

## References

[CR1] Ando T, Kodera N, Takai E, Maruyama D, Saito K, Toda A (2001). A high-speed atomic force microscope for studying biological macromolecules. Proc Natl Acad Sci U S A.

[CR2] Ando T, Uchihashi T, Fukuma T (2008). High-speed atomic force microscopy for nano-visualization of dynamic biomolecular processes. Prog Surf Sci.

[CR3] Ando T, Uchihashi T, Scheuring S (2014). Filming biomolecular processes by high-speed atomic force microscopy. Chem Rev.

[CR4] Bell GI (1978). Models of the specific adhesion of cells to cells. Science.

[CR5] Bezanilla M, Drake B, Nudler E, Kashlev M, Hansma PK, Hansma HG (1994). Motion and enzymatic degradation of DNA in the atomic force microscope. Biophys J.

[CR6] Binnig G (1992). Force microscopy. Ultramicroscopy.

[CR7] Binnig G, Quate CF, Gerber C (1986). Atomic force microscope. Phys Rev Lett.

[CR8] Binnig G, Gerber C, Stoll E, Albrecht TR, Quate CF (1987) Atomic resolution with atomic force microscope. Surf Sci 189–190:1–6. 10.1016/S0039-6028(87)80407-7

[CR9] Butt H-J, Siedle P, Seifert K, Fendler K, Seeger T, Bamberg E, Weisenhorn AL, Goldie K, Engel A (1993). Scan speed limit in atomic force microscopy. J Microsc.

[CR10] Chtcheglova LA, Hinterdorfer P (2018). Simultaneous AFM topography and recognition imaging at the plasma membrane of mammalian cells. Semin Cell Dev Biol.

[CR11] Drake B, Prater CB, Weisenhorn AL, Gould SAC, Albrecht TR, Quate CF, Cannell DS, Hansma HG, Hansma PK (1989). Imaging crystals, polymers, and processes in water with the atomic force microscope. Science.

[CR12] Dufrêne YF, Ando T, Garcia R, Alsteens D, Martinez-Martin D, Engel A, Gerber C, Müller DJ (2017). Imaging modes of atomic force microscopy for application of molecular and cell biology. Nat Nanotechnol.

[CR13] Erie DA, Yang G, Schultz HC, Bustamante C (1994). DNA bending by Cro protein in specific and nonspecific complexes: implications for protein site recognition and specificity. Science.

[CR14] Evans E, Ritchie K (1997). Dynamic strength of molecular adhesion bonds. Biophys J.

[CR15] Florin EL, Moy VT, Gaub HE (1994). Adhesion forces between individual ligand-receptor pairs. Science.

[CR16] Guthold M, Bezanilla M, Erie DA, Jenkins B, Hansma HG, Bustamante C (1994). Following the assembly of RNA polymerase - DNA complexes in aqueous solutions with the scanning force microscope. Proc Natl Acad Sci U S A.

[CR17] Guthold M, Zhu X, Rivetti C, Yang G, Thomson NH, Kasas S, Hansma HG, Smith B, Hansma PK, Bustamante C (1999). Direct observation of one-dimensional diffusion and transcription by Escherichia coli RNA polymerase. Biophys J.

[CR18] Häberle W, Höber JKH, Ohnesorge F, Smith DPE, Binnig G (1992). In situ investigations of single living cells infected by viruses. Ultramicroscopy.

[CR19] Hinterdorfer P, Baumgartner W, Gruber HJ, Schilcher K, Schindler H (1996) Detection and localization of individual antibody-antigen recognition events by atomic force microscopy. Proc Natl Acad Sci U S A 93:3477–3481. 10.1073/pnas.93.8.3477 10.1073/pnas.93.8.3477 PMC39634 8622961

[CR20] Kasas S, Thomson NH, Smith BL, Hansma HG, Zhu X, Guthold M, Bustamante C, Kool ET, Kashlev M, Hansma PK (1997). Escherichia coli RNA polymerase activity observed using atomic force microscopy. Biochemistry.

[CR21] Krieg M, Fläschner G, Alsteens D, Gaub BM, Roos WH, Wuite GJL, Gaub HE, Gerber C, Dufrêne YF, Müller DJ (2019). Atomic force microscopy-based mechanobiology. Nat Rev Phys.

[CR22] Marti O, Drake B, Hansma PK (1987). Atomic force microscopy of liquid-covered surfaces: Atomic resolution images. Appl Phys Lett.

[CR23] Meyer G, Amer NM (1988). Novel optical approach to atomic force microscopy. Appl Phys Lett.

[CR24] Müller DJ, Dufrêne YF (2008). Atomic force microscopy as a multifunctional molecular toolbox in nanobiotechnology. Nat Nanotechnol.

[CR25] Ohnesorge F, Heckl WM, Häberle W, Pum D, Sara M, Schindler H, Schilcher K, Kiener A, Smith DPE, Sleytr UB, Binnin G (1992). Scanning force microscopy studies of the S-layers from Bacillus coagulans E38-66, Bacillus sphaericus CCM2177 and of an antibody binding process. Ultramicroscopy.

[CR26] Puchner EM, Gaub HE (2009). Force and function: probing proteins with AFM-based force spectroscopy. Curr Opin Struct Biol.

[CR27] Rico F, Gonzalez L, Casuso I, Puig-Vidal M, Scheuring S (2013). High-speed force spectroscopy unfolds titin at the velocity of molecular dynamics simulations. Science.

[CR28] Runel G, Lopez-Ramirez N, Chlasta J, Masse I (2021). Biomechanical properties of cancer cells. Cells.

[CR29] Stroh C, Wang H, Bash R, Ashcroft B, Nelson J, Gruber H, Lohr D, Lindsay SM, Hinterdorfer P (2004). Single-molecule recognition imaging microscopy. Proc Natl Acad Sci U S A.

[CR30] Valotteau C, Sumbul F, Rico F (2019). High-speed force spectroscopy: microsecond force measurements using ultrashort cantilevers. Biophys Rev.

[CR31] Vianni MB, Pietrasanta LI, Thompson JB, Chand A, Gebeshuber IC, Kindt JH, Richter M, Hansma HG, Hansma PK (2000). Probing protein-proein interactions in real time. Nat Struct Biol.

[CR32] Weisenhorn AL, Khorsandi M, Kasas S, Gotzos V, Butt H-J (1993). Deformation and height anomaly of soft surfaces studied with an AFM. Nanotechnology.

[CR33] Xu W, Mezencev R, Kim B, Wang L, McDonald J, Sulchek T (2012). Cell stiffness is a biomarker of the metastatic potential of ovarian cancer cells. PLoS One.

[CR34] Yuan C, Chen A, Kolb P, Moy VT (2000). Energy landscape of streptavidin-biotin complexes measured by atomic force microscopy. Biochemistry.

[CR35] Zhong Q, Inniss D, Kjoller K, Elings VB (1993). Fractured polymer/scilica fiber surfaces studied by tapping mode atomic force microscopy. Surf Sci Lett.

[CR36] Zhu R, Howorka S, Pröll J, Kienberger F, Preiner J, Hesse J, Ebner A, Pastushenko VP, Gruber HJ, Hinterdorfer P (2010). Nanomechanical recognition measurements of individual DNA molecules reveal epigenetic methylation patterns. Nat Nanotechnol.

